# THE ISOLATION OF OLDER ADULTS: A COMPARISON OF JAPANESE GENDERS AND HOUSEHOLDS

**DOI:** 10.1093/geroni/igz038.1943

**Published:** 2019-11-08

**Authors:** Keiko Katagiri

**Affiliations:** Kobe University, Kobe, Hyogo, Japan

## Abstract

As the aging population increases, large changes have occurred among household structures in Japan. Half of older adults lived with their children’s families in 1980. Now around 60 percent of them live by themselves: 27% in single households and 31% as older couples alone. Older adults in single households are said to be at higher risk of social isolation. A Japanese white paper reported that they had scanty of social interactions compared to other types of households. This study examines differences in the social relationships and health statuses among household types by gender and explores the risk factors of social isolation. Nationally representative 2012 Japanese Social Survey data were used for analyses; a subsample comprised participants aged 60 to 74 years. A series of ANCOVAs were conducted. The distribution of the gender and household types were single male 105 (10.0%), married male 387 (36.8%), single female 180 (17.1%), and married female 381 (36.2%); the main effects were being female and married. An interaction effect between them (single males were less happy than married males) was observed. Neighborhood relationships were better among females and married participants. Married participants were more active in community meetings, social participation, and volunteering. However, no difference was observed in social network size. Thus, network size alone was not related to social isolation, but being active in social relationships and the quality of relationships influenced social isolation and well-being. Being married and female may facilitate higher quality relationships and may lead to activity and buffer social isolation.

